# Evaluation of P-Glycoprotein Inhibitory Potential Using a Rhodamine 123 Accumulation Assay

**DOI:** 10.3390/pharmaceutics8020012

**Published:** 2016-04-12

**Authors:** Elodie Jouan, Marc Le Vée, Abdullah Mayati, Claire Denizot, Yannick Parmentier, Olivier Fardel

**Affiliations:** 1 Institut de Recherches en Santé, Environnement et Travail (IRSET), UMR INSERM U1085, Faculté de Pharmacie, 2 Avenue du Pr Léon Bernard, 35043 Rennes, France; elodie.jouan@gmail.com (E.J.); marc.levee@free.fr (M.L.V.); abdullah.mayati@univ-rennes1.fr (A.M.); 2 Centre de Pharmacocinétique, Technologie Servier, 25-27 Rue Eugène Vignat, 45000 Orléans, France; claire.denizot@servier.com (C.D.); yannick.parmentier@servier.com (Y.P.); 3 Pôle Biologie, Centre Hospitalier Universitaire, 2 rue Henri le Guilloux, 35033 Rennes, France

**Keywords:** P-glycoprotein, inhibition, drug–drug interactions, rhodamine 123, digoxin

## Abstract

*In vitro* evaluation of P-glycoprotein (P-gp) inhibitory potential is now a regulatory issue during drug development, in order to predict clinical inhibition of P-gp and subsequent drug–drug interactions. Assays for this purpose, commonly based on P-gp-expressing cell lines and digoxin as a reference P-gp substrate probe, unfortunately exhibit high variability, raising thus the question of developing alternative or complementary tests for measuring inhibition of P-gp activity. In this context, the present study was designed to investigate the use of the fluorescent dye rhodamine 123 as a reference P-gp substrate probe for characterizing P-gp inhibitory potential of 16 structurally-unrelated drugs known to interact with P-gp. 14/16 of these P-gp inhibitors were found to increase rhodamine 123 accumulation in P-gp-overexpressing MCF7R cells, thus allowing the determination of their P-gp inhibitory potential, *i.e.*, their half maximal inhibitor concentration (IC_50_) value towards P-gp-mediated transport of the dye. These IC_50_ values were in the range of variability of previously reported IC_50_ for P-gp and can be used for the prediction of clinical P-gp inhibition according to Food and Drug Administration (FDA) criteria, with notable sensitivity (80%). Therefore, the data demonstrated the feasibility of the use of rhodamine 123 for evaluating the P-gp inhibitory potential of drugs.

## 1. Introduction

P-glycoprotein (P-gp), encoded by the multidrug resistance (MDR) 1 gene (also known as *ABCB1*), is an ATP-binding cassette (ABC) membrane transporter acting as a drug efflux pump [1,2]. This membrane protein was initially characterized as overexpressed in tumoral cells exhibiting multiple resistance to a wide variety of structurally-unrelated anticancer drugs [3]. P-gp was next shown to be physiologically expressed in tissues and organs involved in pharmacokinetics, such as intestine, liver, kidney and blood–brain barrier, and to handle drugs belonging to various pharmacological classes. By this way, P-gp plays a notable role in intestinal absorption, brain distribution, and hepatobiliary and/or renal secretion of several marketed drugs, including the cardiotonic drug digoxin and the hypotensive agent talinolol; P-gp can therefore be considered as a key actor of the pharmacokinetics of these compounds [4].

P-gp activity can be inhibited by a wide range of drugs, which can lead to drug–drug interactions (DDIs) due to impairment of pharmacokinetics of a drug substrate for P-gp (the “victim”) by the inhibitor drug (the “perpetrator”). This notably concerns digoxin, for which various clinical DDIs involving P-gp have been reported [5]. Indeed, digoxin has a narrow therapeutic index and even slight changes in plasma exposure due to alteration of P-gp activity can alter its efficacy and/or safety. Owing to this implication of P-gp in DDIs, the determination of P-gp inhibitory potential is now required by drug regulatory agencies such as the US Food and Drug Administration (FDA) and the European Medicines Agency (EMA) for new drugs developed by pharmaceutical companies [6]. In particular, the FDA recommends determining *in vitro* half maximal inhibitor concentration (IC_50_) values towards P-gp and provides decision criteria using these IC_50_ values for evaluating the risk of a clinically significant DDI resulting from P-gp inhibition. A clinical study with digoxin as a P-gp substrate thus must be considered when the maximum total plasma (bound plus unbound) concentration of the investigated drug at steady state ([I_1_]) divided by its *in vitro* P-gp inhibitory potency (IC_50_) is greater than or equal to 0.1 or, for orally administered drugs, its nominal gut concentration ([I_2_]) divided by its IC_50_ towards P-gp is greater than or equal to 10 [7].

Assays for determining *in vitro* P-gp inhibitory potential (IC_50_) are commonly based on P-gp-expressing cell lines, such as Caco-2 cell line and *MDR1*-transfected MDCKII or LLC-PK1 cell lines, and digoxin as a marketed reference, a non-metabolized and sensitive P-gp probe [8,9], for which many clinical DDI data are available [5]. Inside-out P-gp vesicles and *N*-methylquininide or vinblastine as P-gp substrates are alternatively used [8]. Recent data from the “P-gp IC_50_ working group” have, however, revealed a marked inter-laboratory variability in P-gp IC_50_ determinations from digoxin transport assays across polarized cell monolayers or from P-gp vesicles assays [10], resulting in the questioning of the predictability of P-gp-mediated DDIs from *in vitro* data [11]. Moreover, the use of digoxin as a specific P-gp probe in transepithelial efflux assays has been recently challenged because digoxin is handled by (an) as yet unidentified basolateral uptake process(es) in intestinal cells [12]. It also required rather elaborated and specific bioanalytical methods and equipment, *i.e.*, a scintillation counter when using radiolabeled digoxin or a liquid chromatography coupled to tandem mass spectrometry (LC–MS/MS) for quantifying unlabeled digoxin. These concerns about the digoxin assay raise the question of the development of alternative assays for P-gp IC_50_ determination. In this context, fluorescent dye-based assays are likely to be considered, notably because they are relatively easy to use and are applicable to high throughput assays [13]. Among fluorescence dyes transported by P-gp, rhodamine 123 is likely a major one, which has been successfully applied to the detection of P-gp activity in a wide range of studies [13,14,15,16], with a rather high sensitivity when compared to the use of other dyes [17,18]. However, whether a rhodamine 123 assay may allow the evaluation of P-gp inhibitory potential in a convenient and calibrated manner remains to be established. The present study was therefore designed to determine IC_50_ values for 16 reference P-gp inhibitors using a rhodamine 123 accumulation assay and to investigate their potential usefulness for the prediction of P-gp-related clinical relevant DDIs, notably in comparison with IC_50_ data from digoxin transport assays available in the literature.

## 2. Materials and Methods

### 2.1. Chemicals

Rhodamine 123, amiodarone, carvedilol, cyclosporin A, diltiazem, elacridar, felodipine, fumitremorgin C, isradipine, itraconazole, mibefradil, nicardipine, nitrendipine, probenecid, quinidine, sertraline, troglitazone, verapamil, and zosuquidar were provided by Sigma–Aldrich (Saint-Quentin Fallavier, France).

### 2.2. Cell Culture

Parental human mammary MCF7 cells and P-gp-overexpressing MCF7R cells [19], generated by stepwise selection with doxorubicin [20], were cultured in Dulbecco’s modified Eagle medium (DMEM) (Life Technologies, Saint-Aubin, France), supplemented with 10% (*v*/*v*) fetal calf serum, 100 IU/mL penicillin, and 100 μg/mL streptomycin.

### 2.3. RNA Isolation and Analysis

Total RNAs were extracted using the TRI reagent (Sigma–Aldrich). RNA was then subjected to a reverse transcription-quantitative polymerase chain reaction (RT-qPCR), using the RT kit from Applied Biosystems (Foster City, CA, USA), the fluorescent dye SYBR Green methodology, and an ABI Prism 7300 detector (Applied Biosystems), as described previously [21]. Gene-specific primers for drug transporters and 18S RNA were used exactly as already reported [22]. The specificity of each gene amplification was verified at the end of qPCR reactions through analysis of dissociation curves of the PCR products. Amplification curves were analyzed with ABI Prism 7000 SDS software, using the comparative cycle threshold method. Relative quantification of the steady-state target mRNA levels was calculated after normalization of the total amount of cDNA tested to the 18S RNA endogenous reference using the 2^(−Δ*C*t)^ method. Data were finally expressed in arbitrary units relative to 18S RNA content as described previously [23].

### 2.4. Western Blot Analysis

Cellular protein extracts were prepared as already described [24]. Protein lysates were then separated on polyacrylamide gel and electrophoretically transferred onto Protan^®^ nitrocellulose membranes (Whatman GmbH, Dassel, Germany). After blocking with Tris-buffered saline containing 4% (*v*/*v*) bovine serum albumin for 30 min at room temperature, membranes were incubated overnight at 4 °C with primary antibodies against P-gp (clone C219) (Alexis Biochemicals, Lausen, Switzerland) or HSC70 (Santa Cruz Biotechnology, CA, USA), used here as a control for gel loading and transfer. After washing, membranes were next re-incubated with appropriate horseradish peroxidase-conjugated secondary antibodies (Dako, Glostrup, Denmark). Immunolabeled proteins were finally visualized by chemiluminescence.

### 2.5. Rhodamine 123 Accumulation Assay

P-gp activity was determined by measuring intracellular accumulation of rhodamine 123 in MCF7R cells in the absence or presence of P-gp inhibitors, as previously reported [25]. Briefly, for a typical IC_50_ determination experiment, cells were incubated at 37 °C with 5.25 μM rhodamine 123 for 30 min, in the presence or absence of various concentrations of P-gp inhibitors. After washing in phosphate-buffered saline, cells were lysed in distilled water, and intracellular levels of rhodamine 123 were quantified by spectrofluorimetry using a SpectraMax Gemini SX spectrofluorimeter (Molecular Devices, Sunnyvale, CA, USA) (excitation and emission wavelengths were 485 and 535 nm, respectively). Data were expressed as % of rhodamine 123 accumulation in control cells not exposed to P-gp inhibitors, arbitrarily set at 100%. IC_50_ values for inhibition of P-gp activity, which correspond to half maximal effective concentration (EC_50_) values for increasing rhodamine 123 accumulation, were next determined by 4 parameter logistic non-linear regressions using the Prism software (GraphPad software, La Jolla, CA, USA).

### 2.6. Data Analysis

Experimental data were routinely expressed as means ± SEM from at least three independent experiments. Statistical analysis of quantitative data was performed with a Student's *t*-test, analysis of variance (ANOVA), followed by Dunnett’s *post-hoc* test or non-parametric Spearman rank correlation using the Prism software. The criterion of significance was *p* < 0.05.

Analysis of [I_1_]/IC_50_ and [I_2_]/IC_50_ ratio in relation to relevance or non relevance of clinical DDI studies dealing with P-gp and digoxin was performed using a binary classification decision tree, given a fixed discrimination threshold, resulting in four possible outcomes [26]: true positive (TP)—*in vitro* data is in agreement with a relevant clinical digoxin DDI; false negative (FN)—*in vitro* data is not in agreement with a relevant clinical digoxin DDI; false positive (FP)—*in vitro* data is not in agreement with a non relevant clinical digoxin DDI; and true negative (TN)—*in vitro* data is in agreement with a non relevant clinical digoxin DDI. The following performance metrics were next determined using the following equations:

(1) Accuracy (%) = (Number of TP + Number of TN) × 100 / Total number of studies

(2) Sensitivity (%) = Number of TP × 100 / (Number of TP + Number of FN)

(3) Specificity (%) = Number of TN × 100 / (Number of TN + Number of FP)

## 3. Results and Discussion

### 3.1. Expression of MDR1/P-gp in MCF7 and MCF7R Cells

**Figure 1:**
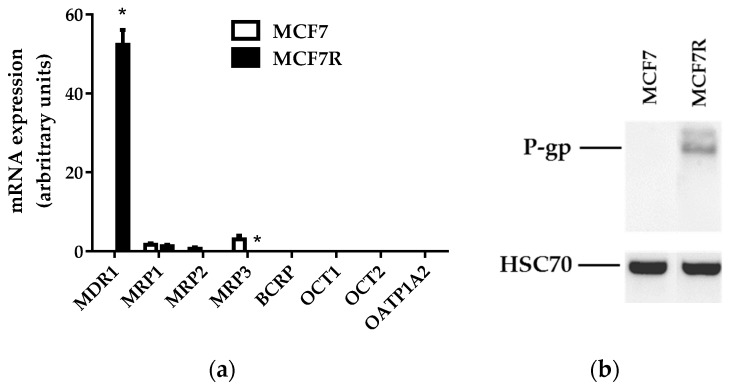
*MDR1* mRNA and P-gp expression in MCF7 and MCF7R cells. (**a**) Drug transporter mRNA expression determined by RT-qPCR. Data are expressed as arbitrary units and are the means ± SEM of three independent assays. *, *p* < 0.05 when compared to transporter mRNA expression found in MCF7 cells. (**b**) P-gp expression analyzed via Western blot. Data shown are representative of three independent assays.

We first characterized the expression of *MDR1* mRNAs and P-gp in MCF7R cells, used as reference P-gp-overexpressing cells in our rhodamine accumulation assay. These cells were found to markedly overexpress *MDR1* mRNAs (Figure 1a) and P-gp (Figure 1b) when compared to parental MCF7 cells. By contrast, MCF7R cells, as well as MCF7 cells, exhibited no detectable mRNA expression of other transporters that have been demonstrated to handle rhodamine 123 such as mutated breast cancer resistance protein (BCRP/*ABCG2*) [27], organic cation transporter (OCT) 1 (*SLC22A1*), OCT2 (*SLC22A2*) [28], and organic anion-transporting polypeptide (OATP) 1A2 (*SLCO1A2*) [29] (Figure 1a). With respect to mRNA expression of additional ABC efflux pumps, such as multidrug resistance-associated protein (MRP) 1 (*ABCC1*), MRP2 (*ABCC2*), and MRP3 (*ABCC3*), MCF7 and MCF7R cells exhibited similarly low expression of MRP1, whereas MRP2 and MRP3 were not expressed (MCF7R cells) or only poorly expressed (MCF7 cells) (Figure 1a). MCF7R cells thus clearly showed overexpression of P-gp when compared to MCF7 cells, without notable expression of other transporters, which fully justifies the use of these cells for specifically studying P-gp activity.

### 3.2. Rhodamine 123 Accumulation in MCF7 and MCF7R Cells

**Figure 2:**
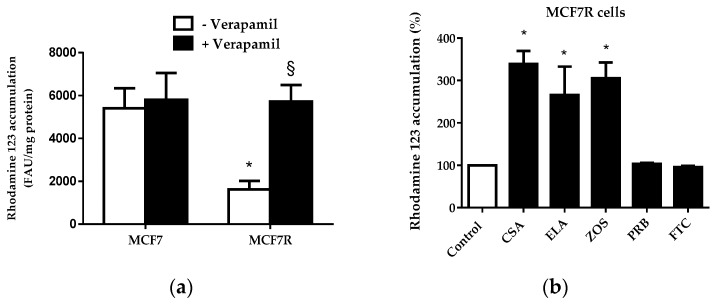
Accumulation of rhodamine 123 in MCF7 and MCF7R cells. (**a**) Cells were exposed to 5.25 µM rhodamine 123 for 30 min at 37 °C in the absence or presence of 50 µM verapamil. After washing, rhodamine 123 accumulation was quantified by spectrofluorimetry. Data are expressed as fluorescence arbitrary unit (FAU)/mg protein and are the means ± SEM of three independent experiments. *, *p* < 0.05 when compared to MCF7 cells; §, *p* < 0.05 when compared to counterparts not exposed to verapamil. (**b**) MCF7R cells were exposed to 5.25 µM rhodamine 123 for 30 min at 37 °C in the absence (control) or presence of 100 µM cyclosporin A (CSA), 1 µM elacridar (ELA), 1 µM zosuquidar (ZOS), 2 mM probenecid (PRB) or 10 µM fumitremorgin C (FTC). After washing, cellular rhodamine 123 accumulation was quantified by spectrofluorimetry. Data are expressed as % of fluorescent dye accumulation in control MCF7R cells exposed only to rhodamine 123, arbitrarily set at 100%, and are the means ± SEM of three independent assays. *, *p* < 0.05 when compared to control cells.

As shown in Figure 2a, MCF7R cells poorly accumulated rhodamine 123 when compared to parental MCF7 cells, thus reflecting P-gp-mediated efflux of the fluorescent dye in MCF7R cells. In the presence of the reference P-gp inhibitor verapamil, used at a 50-µM concentration known to fully inhibit P-gp activity [30], rhodamine 123 accumulation in MCF7R cells was restored to the level found in MCF7 cells (Figure 2a); by contrast, verapamil did not alter rhodamine 123 level in MCF7 cells. In addition to verapamil, other reference P-gp inhibitors such as cyclosporin A [31], elacridar [32], and zosuquidar [33] increased dye accumulation in MCF7R cells (Figure 2b). By contrast, probenecid and fumitremorgin C, reference inhibitors for MRPs and BCRP, respectively [34,35], failed to augment rhodamine 123 levels in MCF7R cells (Figure 2b). Taken together, these data demonstrate that the use of the rhodamine 123 assay was adequate for specifically measuring P-gp activity and its inhibition by reference P-gp inhibitors in MCF7R cells. This conclusion is, moreover, reinforced by the fact that rhodamine 123 is not a substrate for cytochrome P-450 3A [36], in contrast to many other P-gp substrates [37], thus discarding the hypothesis that the modulation of cellular rhodamine 123 accumulation by some P-gp inhibitors may be due to interference with cytochrome P-450 3A activity. It should, however, be kept in mind when interpreting data from rhodamine 123 transport assay that the dye can be partly metabolized to rhodamine 110 through deacetylation followed by its glucuronidation [38]. Moreover, the fact that mitochondrial membrane potential is an additional factor contributing to rhodamine 123 accumulation [39] must also be formally taken into account. In this context, however, it is noteworthy that drugs interfering with mitochondria usually decrease, and not increase, mitochondrial transmembrane potential [40], thus allowing easy discrimination of drugs inhibiting P-gp (that increase cellular accumulation of rhodamine 123 in P-gp expressing cells through inhibiting its efflux) from drugs interacting with mitochondria (that reduce cellular accumulation of rhodamine 123 through lowering mitochondrial transmembrane potential).

### 3.3. Determination of P-gp IC_50_ Values for a Range of Structurally-Unrelated Reference P-gp Inhibitors

**Figure 3:**
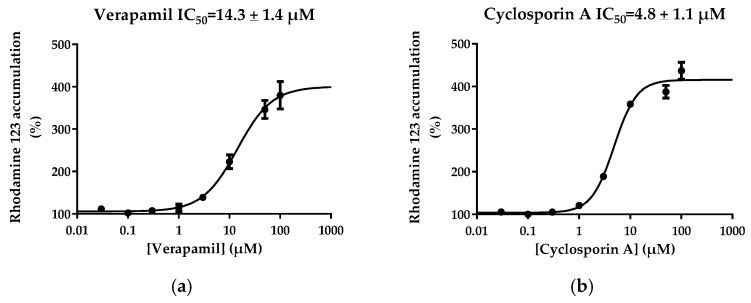
Concentration-dependent effects of verapamil and cyclosporin A on rhodamine 123 accumulation in MCF7R cells. Cells were exposed to 5.25 µM rhodamine 123 for 30 min at 37 °C in the absence or presence of various concentrations of (**a**) verapamil or (**b**) cyclosporin A. After washing, rhodamine 123 accumulation was quantified by spectrofluorimetry. Data are expressed as % of rhodamine 123 levels found in control MCF7R cells not exposed to verapamil or cyclosporin A, arbitrarily set at 100% and are the means ± SEM of three independent experiments. IC_50_ values for P-gp activity, which correspond to EC_50_ values for increase of rhodamine 123 accumulation, are indicated at the top of the graphs.

Concentration-dependent effects of 16 structurally-unrelated reference P-gp inhibitors on cellular accumulation of rhodamine 123 were determined in MCF7R cells. These inhibitors, listed in Table 1, have been well characterized previously for their inhibitory potential towards P-gp-mediated transport of digoxin both *in vitro* [5,10,41] and in the clinic [41,42]. Among these 16 chemicals, 14 were found to inhibit P-gp-mediated efflux of rhodamine 123 in MCF7R cells, *i.e.*, they increased cellular accumulation of rhodamine 123 (Table 1). These effects were concentration-dependent; examples of curves for increased rhodamine 123 accumulation *versus* inhibitor concentrations are shown in Figure 3 for verapamil and cyclosporin A. IC_50_ values towards P-gp activity were finally determined for each active P-gp inhibitor by non-linear regression of rhodamine 123 assay data (Table 1). These IC_50_ values were found to range from 0.05 µM (for elacridar) to 250.5 µM (for nitrendipine).

Two compounds, *i.e.*, itraconazole and sertraline, were found not to increase rhodamine 123 accumulation in MCF7R cells when used up to 100 µM, thus suggesting that they do not inhibit P-gp-mediated efflux of rhodamine 123 (Table 1). These compounds have, however, been shown to inhibit P-gp-mediated efflux of digoxin *in vitro* [41]; moreover, itraconazole can block clinical P-gp activity in humans [43,44], whereas, by contrast, inhibition of P-gp activity by sertraline observed *in vitro* is unlikely to be of major clinical significance [45]. Interestingly, itraconazole has been shown to block P-gp-mediated efflux of the anticancer drug daunorubicin or of acetoxymethyl ester of the dye calcein, but not that of rhodamine 123 in *MDR1*-transfected cells or in intestinal Caco-2 cells [46,47]. The reasons for such differences with respect to P-gp activity inhibition according to substrates are likely due to the presence of multiple drug binding sites on P-gp, with which P-gp substrates and inhibitors differently interact [47]. The best characterized sites most likely correspond to the H-site (binding Hoechst 33482), the R-site (binding rhodamine 123), and the P-site (binding prazosine and progesterone) [48,49]. In this context, itraconazole and sertraline may be presumed to not, or only poorly, interact with the R-site of P-gp, which suggests that the use of rhodamine 123 as a P-gp substrate fails to detect some P-gp inhibitors. Inhibition of P-gp by drugs is therefore likely substrate-dependent [50]. The use of different substrates interacting specifically with the different drug binding sites of P-gp should therefore be considered theoretically in order to accurately characterize the putative P-gp inhibitory potential of a given drug. This would require detailed knowledge of the specific P-gp substrate binding site involved in drug binding, which unfortunately is not available yet—not even for relevant P-gp substrates like digoxin. In addition, a fourth site on P-gp, which may bind non-transported modulators like elacridar or nicardipine [51], must also be considered. This site has been hypothesized to be a regulatory site, interacting with other sites in an allosteric manner [51], thus likely explaining why elacridar and nicardipine inhibit transport of rhodamine 123 (Table 1), presumably without binding to the R-site.

### 3.4. Comparison of IC_50_ Values for P-gp Inhibition Determined from Rhodamine 123 and Digoxin Transport Assays

**Figure 4:**
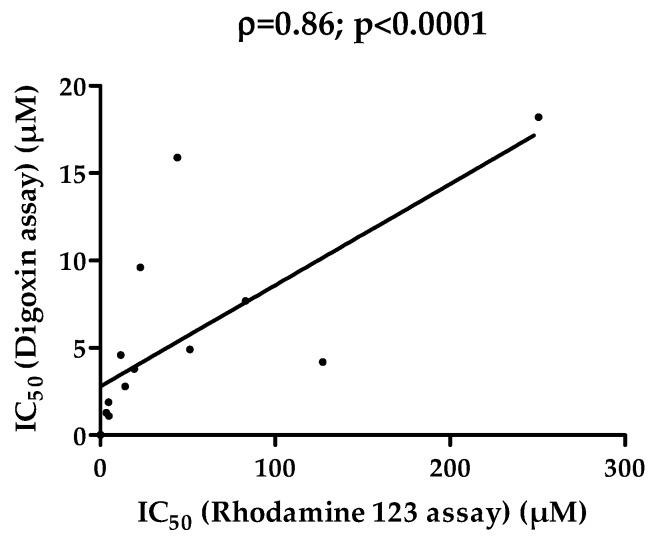
Correlation of IC_50_ values for P-gp inhibition from rhodamine 123 assay and digoxin assay. IC_50_ values towards P-gp activity for 14 drugs listed in Table 1, determined from rhodamine 123 accumulation assays and from digoxin bidirectional transport assays reported by Poirier *et al.* [41], were analyzed via the Spearman rank correlation method. Spearman coefficient (ρ) and *p* value are provided at the top of the correlation graph.

Next, IC_50_ values for 14 P-gp inhibitors measured by the rhodamine 123 assay (Table 1) were compared to IC_50_ values determined by the digoxin bidirectional transport assay in *MDR1*-transfected cells as reported by Poirier *et al.* [41]. IC_50_ values for P-gp-mediated inhibition of rhodamine 123 transport were higher than those from digoxin transport assays. This may be due to the nature of the final parameter obtained for generating IC_50_ from digoxin assays, *i.e.*, the digoxin efflux ratio (ER). Indeed, this parameter, corresponding to the ratio of digoxin apparent permeability in the basolateral-to-apical direction (P_app_BA) *versus* that in the apical-to-basolateral direction (P_app_AB), has been shown to be particularly sensitive to P-gp inhibitors, much more than the parameters P_app_AB or P_app_BA. IC_50_ values based on digoxin P_app_AB or P_app_BA for a wide range of P-gp inhibitors were thus higher than those generated from digoxin ER [41], which may be due to the fact that the influence of passive permeability is maximally neglected with the ER parameter [52]. Interestingly, the ratio “IC_50_ rhodamine 123 assay”/“IC_50_ digoxin assay”, which can be considered as the fold-variability, ranged from 1.9-fold (for elacridar) to 30.3-fold (for felodipine) (Table 1). They remained, thus, for each of the 14 P-gp inhibitors active on rhodamine 123 transport, in the IC_50_ range established from five experimental measurements based on digoxin transport and vesicle uptake of P-gp substrates [11] (Table 1). Such data, therefore, underline the potential relevance of the rhodamine 123 accumulation assay for evaluating the P-gp inhibitory potential. In this context, the rather extended IC_50_ range for P-gp inhibitors, reflecting the now well-established high level of variability for P-gp inhibitory potential characterization [11], deserves attention. The reasons for discrepancies between determinations for a specific P-gp inhibitor remain to be unequivocally specified; they could be linked to various causes, which, moreover, can add up. Differences in the cell type used for *in vitro* assays, which may differentially express additional non-P-gp transporters interacting with P-gp substrates such as digoxin [12,53] or may constitutively express non-human P-gp when they are of animal origin [54], may be suspected. The nature of the final measured parameter, *i.e.*, for example, intracellular accumulation of rhodamine 123 in P-gp-overexpressing MCF7R cells (in the present study) *versus* ER of digoxin across monolayers of *MDR1*-transfected cells (in the study of Poirier *et al.* [41]), may also be incriminated. Additionally, IC_50_ calculation methods as well as inter-laboratory variability should be considered, as discussed recently [10]. Nevertheless, it is noteworthy that IC_50_ values from rhodamine 123 assay showed highly significant correlation with those from the digoxin assay [41] (Figure 4), indicating that the two methods similarly ranked the P-gp inhibitors according to their inhibitory potential.

### 3.5. Prediction of in Vivo P-gp Inhibition from Rhodamine 123 Accumulation Assays

To analyze the putative relevance of IC_50_ values generated from rhodamine 123 accumulation for predicting clinical P-gp inhibition, we considered 26 clinical studies, involving digoxin as a victim drug and some of the 16 P-gp inhibitors investigated in the present study as perpetrator drugs. These studies were retrieved from 68 published studies listed by Poirier *et al.* [41]. Their clinical data, including plasma [I_1_] or intestinal [I_2_] concentrations of the perpetrator, have been detailed by Poirier *et al.* [41] and are summarized in Table 2. Twenty out of 26 studies described relevant DDI, *i.e.*, the ratio area under the curve in the presence of the P-gp inhibitor (AUCi) *versus* that in the absence of the inhibitor (AUC) and/or the ratio peak plasma concentration in the presence of the P-gp inhibitor (*C*_max_i) *versus* that in absence of inhibitor (*C*_max_) were ≥ 1.25. These thresholds are thought to be significant regarding the toxicity of digoxin [5] and have consequently been retained in various previous studies [5,41,42,52]. The remaining 6/26 studies were classified as non relevant DDI (ratio AUCi/AUC < 1.25 and ratio *C*_max_i/*C*_max_ < 1.25) [41].

Further, the ratio [I_1_]/IC_50_ and [I_2_]/IC_50_ were calculated with IC_50_ values determined from the rhodamine 123 assay for each DDI study (data not shown) and were used to predict P-gp-related DDI for the 26 clinical studies according to FDA criteria ([I_1_]/IC_50_ > 0.1 and/or [I_2_]/IC_50_ > 10). Prediction results for the 26 clinical studies are indicated in Table 2. For the 20 clinically relevant DDI, 16 were found to be predicted correctly with IC_50_ values from rhodamine 123 assays and FDA criteria (Table 2), thus resulting in a rather high sensitivity (80.0%) (Table 3). Omitting itraconazole, which does not interact with the R-site of P-gp as discussed above, the sensitivity even reaches 84.2%, in the range of sensitivity values (around 80%–90%) previously reported for prediction of clinical P-gp inhibition [26,41,42]. By contrast, for the 6 studies with non-relevant DDI, only one was correctly predicted with IC_50_ values from the rhodamine 123 assay (Table 2), thus leading to a rather low specificity (16.7%) and an intermediate rate of accuracy (65.4%) (Table 3). Interestingly, using FDA criteria and IC_50_ values from digoxin ER assays [41] instead of those based on rhodamine 123 assays, similar predictions of clinical P-gp inhibition were obtained (Table 2), with, however, slightly higher sensitivity (95.0%) and accuracy (80.0%) and similarly low specificity (16.7%) (Table 3). This low specificity for prediction of clinical P-gp inhibition, due to a high rate of false positives found with IC_50_ values from the rhodamine 123 or the digoxin assays, should be interpreted with caution, because of the small number of negative clinical DDI studies (*n* = 6) in the present study. However, it is noteworthy that a high rate of false positives (51%) has also been reported in previous predictions of clinical P-gp inhibition with a much larger clinical data set (*n* = 101) [42]. This has been hypothesized to be related to the inappropriate nature of [I_2_] criterion proposed by the FDA, for which a clinical digoxin study becomes warranted for many drugs with a quantifiable IC_50_ [11].

The high rate of false positives for prediction of clinical P-gp inhibition from *in vitro* data has led to the questioning of FDA thresholds [11] and to apply receiver operator characteristic analysis for establishing new criteria [41,42]. The “P-gp IC_50_ working group”, established with 23 participating pharmaceutical and contract research laboratories and one academic institution [10], has thus proposed to retain the ratio [I_1_]/IC_50_ > 0.03 and/or [I_2_]/IC_50_ > 45 [42] for predicting *in vivo* relevant P-gp interaction, whereas the threshold considered by Poirier *et al.* [41] refers only to intestinal concentration ([I_2_]/IC_50_ > 6.5). Applying the “P-gp IC_50_ working group” criteria to IC_50_ values generated from rhodamine 123 accumulation assays allowed the enhancement of specificity and accuracy to 50% and 73.1% (Table 4), respectively, whereas sensitivity (80%) remained constant when compared to the use of FDA criteria. By contrast, the application of the criteria proposed by Poirier *et al.* [41] failed to enhance specificity and accuracy (Table 4).

The rather notable sensitivity of the rhodamine 123 assay for predicting clinical P-gp interactions may be compatible with its use for *in vitro* screening of the P-gp inhibitory potential of drug candidates during their pharmaceutical development. However, limits of the rhodamine 123 assay already discussed above, notably its failure to detect P-gp inhibitors not interacting with the R-site of P-gp and the P-gp-unrelated factors that may affect cellular accumulation of the dye, must be kept in mind. In fact, an ideal experimental *in vitro* approach for predicting clinical P-gp inhibition may require the use of several P-gp assays in combination, with multiple P-gp substrates, as already suggested [47,50]. Rhodamine 123 assay may be one of these assays, especially convenient for drug screening and early development. Digoxin-based assay is also one major P-gp assays that must be considered in this context. Indeed, extensive data on clinical interactions of P-gp with digoxin are available in humans [5], allowing the close linking of *in vitro* behavior to *in vivo* pharmacokinetics behavior. Moreover, digoxin is recommended by the FDA as a reference P-gp substrate for *in vivo* clinical drug interaction studies [7].

## 4. Conclusions

The fluorescent dye rhodamine 123 was demonstrated to be convenient for characterizing the P-gp inhibitory potential of various reference P-gp inhibitors. As an exception, itraconazole and sertraline produce a negative result in the rhodamine 123 assay and are assumed not to interact with the R-site of P-gp. For drugs inhibiting efflux of rhodamine 123, IC_50_ values towards transport of the dye, although higher than some available IC_50_ towards P-gp-mediated digoxin efflux of P-gp, were in the range of variability of previous reported IC_50_ for P-gp. Moreover, they can be used for the prediction of clinical P-gp inhibition according to FDA criteria, with a rather notable sensitivity (80%). The findings indicate the suitability of the rhodamine 123-based assay for *in vitro* prediction of the clinical risk of interactions with P-gp, notably during drug screening and early development. However, the P-gp inhibitory potential of drugs not interacting with the R-site of P-gp is likely to be complementarily investigated using additional substrates of the pump, thus confirming that accurate prediction of clinically significant P-gp drug interactions require the use of multiple P-gp substrates [50].

## Figures and Tables

**Table 1:** P-gp inhibitory potential (IC_50_ values) based on rhodamine 123 accumulation assay.

Drug	IC_50_ (µM) (Rhodamine 123) ^1^	IC_50_ (µM) (Digoxin) ^2^	Ratio IC_50_ Rhodamine 123/IC_50_ Digoxin	IC_50_ Range for P-gp Inhibition (-fold) ^3^
Amiodarone	22.9 ± 2.0	9.6	2.4	27
Carvedilol	3.4 ± 1.2	1.3	2.7	168
Cyclosporin A	4.8 ± 1.1	1.1	4.4	NR ^4^
Diltiazem	11.7 ± 2.2	4.6	2.6	76
Elacridar	0.05 ± 0.01	0.03	1.9	NR
Felodipine	127.3 ± 6.0	4.2	30.3	188
Isradipine	83.1 ± 10.8	7.7	10.8	24
Itraconazole	No inhibition ^5^	1.8	NA ^6^	NR
Mibefradil	4.7 ± 1.8	1.9	2.5	48
Nicardipine	19.4 ± 1.5	3.8	5.1	78
Nitrendipine	250.5 ± 43.8	18.2	13.8	72
Quinidine	51.3 ± 1.5	4.9	10.5	98
Sertraline	No inhibition ^5^	12.9	NA	18
Troglitazone	44.1 ± 7.7	15.9	2.8	36
Verapamil	14.3 ± 1.4	2.8	5.1	796
Zosuquidar	0.18 ± 0.12	0.02	9.7	NR

^1^ expressed as mean ± SEM of three independent assays; ^2^ data from Poirier *et al.* [41]; ^3^ corresponds to the ratio of the highest *versus* lowest IC_50_ values determined from five experimental measurements based on digoxin transport and vesicle uptake of P-gp substrates, according to Lee *et al.* [11]; ^4^ NR, not reported; ^5^ no inhibition at 100 µM; ^6^ NA, not applicable.

**Table 2:** Prediction of P-gp inhibition in 26 clinical DDI studies with digoxin as a victim drug, using FDA criteria and P-gp IC_50_ values from rhodamine 123 accumulation or digoxin bidirectional transport [41] assays.

Perpetrator	[I_1_]; [I_2_] (µM) ^1,2^	AUCi/AUC ^2,3^	C_max_i/C_max_^2,4^	Clinical Relevance ^5^	P-gp Inhibition Prediction (FDA Criteria ^6^)	
					Rhodamine 123 IC_50_	Digoxin IC_50_^2^
Carvelidol	0.13; 62	1.24	1.00	−	+ (FP) ^7^	+ (FP)
Diltiazem	0.7; 868	1.24	1.24	−	+ (FP)	+ (FP)
Mibefradil	0.8; 404	1.08	1.22	−	+ (FP)	+ (FP)
Nicardipine	0.18; 250	1.06	1.06	−	+ (FP)	+ (FP)
Nitrendipine	0.015; 111	1.09	1.22	−	− (TN) ^8^	− (TN)
Troglitazone	3.62; 3,624	1.04	1.05	−	+ (FP)	+ (FP)
Amiodarone	2.49; 2479	1.63	1.72	+	+ (TP) ^9^	+ (TP)
Amiodarone	2.20; 4959	1.68	1.84	+	+ (TP)	+ (TP)
Carvelidol	0.52; 246	1.2	1.60	+	+ (TP)	+ (TP)
Carvelidol	0.13; 62	1.56	1.38	+	+(TP)	+(TP)
Cyclosporin A	2.8; 1167	NR ^10^	1.44	+	+ (TP)	+ (TP)
Diltiazem	0.7; 579	1.44	1.38	+	+ (TP)	+ (TP)
Diltiazem	0.17; 579	1.51	1.37	+	+(TP)	+(TP)
Felodipine	0.03; 104	1.18	1.34	+	− (FN)^11^	+ (TP)
Isradipine	0.02; 54	1.11	1.26	+	− (FN)	− (FN)
Itraconazole	0.95; 1134	1.68	1.34	+	− (FN)	+ (TP)
Mibefradil	2.42; 1211	1.31	1.41	+	+ (TP)	+ (TP)
Mibefradil	1.61; 807	1.07	1.25	+	+ (TP)	+ (TP)
Nitrendipine	0.03; 222	1.15	1.57	+	− (FN)	+ (TP)
Quinidine	3.54; 3397	1.76	1.75	+	+ (TP)	+ (TP)
Quinidine	5.10; 2466	2.65	NR	+	+ (TP)	+ (TP)
Quinidine	4.5; 2466	NR	1.44	+	+ (TP)	+ (TP)
Verapamil	1.2; 704	1.51	1.44	+	+ (TP)	+ (TP)
Verapamil	0.026; 704	NR	1.53	+	+ (TP)	+ (TP)
Verapamil	0.058; 1056	NR	1.61	+	+ (TP)	+ (TP)
Verapamil	0.033; 704	NR	1.77	+	+ (TP)	+ (TP)

^1^ [I_1_]: total plasma concentration; [I_2_]: gut concentration; ^2^ data from Poirier *et al.* [41]; ^3^ area under the curve in the presence (AUCi) or absence (AUC) of P-gp inhibitor. ^4^ peak plasma concentration in the presence (*C*_max_i) or absence (*C*_max_) of P-gp inhibitor. ^5^ clinical relevance: AUCi/AUC ≥ 1.25 and/or *C*_max_i/*C*_max_ ≥ 1.25; ^6^ FDA criteria: [I_1_]/IC_50_ > 0.1 and/or [I_2_]/IC_50_ > 10; ^7^ FP: false positive; ^8^ TN: true negative; ^9^ TP: true positive; ^10^ NR: not reported; ^11^ FN: false negative.

**Table 3:** Accuracy, sensitivity, and specificity of the prediction of clinical P-gp interactions with IC_50_ values generated from rhodamine 123 accumulation or digoxin bidirectional transport [41] assays, according to FDA criteria ^1^.

Performance Metrics	Prediction from Rhodamine 123 Accumulation Assay	Prediction from Digoxin Transport Assay
Accuracy (%) ^2^	65.4	76.9
Sensitivity (%) ^2^	80.0	95.0
Specificity (%) ^2^	16.7	16.7

^1^ FDA criteria: [I_1_]/IC_50_ > 0.1 and/or [I_2_]/IC_50_ > 10; ^2^ values were generated from the predictions of clinical P-gp interaction for the 26 clinical DDI studies summarized in Table 2 according to FDA criteria.

**Table 4:** Accuracy, sensitivity, and specificity of the prediction of clinical P-gp interactions with IC_50_ values generated from rhodamine 123 accumulation assays according to “P-gp IC_50_ working group” [42] or Poirier *et al.* [41] criteria.

Performance Metrics	Prediction with “P-gp IC_50_ Working Group” Criteria	Prediction with Poirier *et al.* Criteria
Accuracy (%) ^1^	73.1	65.4
Sensitivity (%) ^1^	80.0	80.0
Specificity (%) ^1^	50.0	16.7

^1^ Values were generated from the predictions of clinical P-gp interaction for the 26 clinical DDI studies summarized in Table 2 according to “P-gp IC_50_ working group” criteria ([I_1_]/IC_50_ > 0.03 and/or [I_2_]/IC_50_ > 45) [42] or to criteria by Poirier *et al.* ([I_2_]/IC_50_ > 6.5) [41].
